# Fusion of Infrared and Visible Images Based on Three-Scale Decomposition and ResNet Feature Transfer

**DOI:** 10.3390/e24101356

**Published:** 2022-09-24

**Authors:** Jingyu Ji, Yuhua Zhang, Yongjiang Hu, Yongke Li, Changlong Wang, Zhilong Lin, Fuyu Huang, Jiangyi Yao

**Affiliations:** 1 Department of UAV, Army Engineering University, Shijiazhuang 050003, China; 2 Department of Electronic and Optical Engineering, Army Engineering University, Shijiazhuang 050003, China; 3 Equipment Simulation Training Center, Army Engineering University, Shijiazhuang 050003, China

**Keywords:** infrared and visible image fusion, three-scale decomposition, optimized WLS, ResNet-feature transfer, weighted average strategy

## Abstract

Image fusion technology can process multiple single image data into more reliable and comprehensive data, which play a key role in accurate target recognition and subsequent image processing. In view of the incomplete image decomposition, redundant extraction of infrared image energy information and incomplete feature extraction of visible images by existing algorithms, a fusion algorithm for infrared and visible image based on three-scale decomposition and ResNet feature transfer is proposed. Compared with the existing image decomposition methods, the three-scale decomposition method is used to finely layer the source image through two decompositions. Then, an optimized WLS method is designed to fuse the energy layer, which fully considers the infrared energy information and visible detail information. In addition, a ResNet-feature transfer method is designed for detail layer fusion, which can extract detailed information such as deeper contour structures. Finally, the structural layers are fused by weighted average strategy. Experimental results show that the proposed algorithm performs well in both visual effects and quantitative evaluation results compared with the five methods.

## 1. Introduction

Image fusion plays an important role in many fields, including the medical field, agricultural field, military field, etc. The main purpose of image fusion is to combine the feature information of images captured by multiple sensors into a single image to obtain a rich and comprehensive image, which plays a key role in subsequent image processing tasks [1]. Since infrared and visible image fusion has a wide range of applications, it is the most common type of fusion method. Generally, visible sensors can obtain images with rich detailed information, but they cannot obtain images with rich feature information when there are obstructions, such as smoke or insufficient illumination; infrared sensors can obtain images with rich texture details and other thermal information, and it can still work well in the case of poor brightness, so it can make up for the missing information in the visible image. Infrared image processing is widely used. For example, Zhao et al. [2] addressed the problem of pedestrian detection by placing more emphasis on the underlying temperature information in infrared images. Arora et al. [3] proposed a novel infrared image-correlated data processing method to obtain isothermal patterns from reconstructed pulse-compressed data via a matched filter scheme to identify subsurface anomalies. It can be seen that infrared images can also express a lot of information. Therefore, it is necessary to fuse the infrared image and the visible image.

In recent years, with the continuous development of image fusion technology, various image fusion algorithms emerged one after another. These fusion algorithms roughly include multi-scale transformation-based algorithms, sparse representation-based algorithms, deep learning-based methods, and hybrid methods. The method based on multi-scale transformation is to decompose the image into multi-layer representations, and then use different fusion rules to fuse each layer. This method can extract more detailed features of the source image. From the initial use of Laplace pyramids to describe images [4], later Toet proposed an image fusion method based on contrast pyramids [5] and an image fusion method based on morphological pyramids [6], gradually developing pyramid transformation methods in the field of image fusion. Subsequently, various methods based on multi-scale decomposition continued to appear, and image fusion algorithms based on multi-scale transformation were gradually developed. Long et al. [7] proposed an image fusion algorithm using infrared feature decomposition and obtained a good performance. Kumar et al. [8] proposed a framework that fused the advantages of wavelet transform and sparse representation. Through experiments, it was found that this method overcame the defects of the two methods used alone, and made the fusion result closer to the expected effect. In order to further solve the problem of noise in the fused image, Ma [9] et al. proposed a multi-scale decomposition image fusion method by combining the rolling guided filter and Gaussian filter, and achieved good results. To further achieve scale separation, Li et al. [10] proposed a novel image fusion algorithm utilizing latent low-rank matrix factorization, which is able to extract more salient features from source images. Most of the methods based on multi-scale transformation decompose the image into two parts, which can have certain advantages in extracting detailed features, but there is still a lot of room for improvement.

The method based on sparse representation obtains the fusion image by constructing a sparse decomposition model and reconstructing the dictionary. Yang et al. [11] combined attention visual guidance and sparse representation to obtain sparse coefficients to reconstruct fused images. Liu et al. [12] proposed an image fusion framework that combined multi-scale transformation and sparse representation. By combining the advantages of the two methods, an image fusion algorithm that can adapt to many types of images was obtained. In order to fully retain the image detail information and edge information of the source image, Guo et al. [13] proposed a weighted sparse representation fusion algorithm. The experimental results showed that its fusion effect was better than other algorithms. Although the algorithm based on sparse representation works well in some application scenarios, it is attributed to relying on dictionary learning, so the follow-up research is also replaced by other methods.

With the development of deep learning, it also shows unique advantages in the field of image fusion. Li et al. [14] decomposed the source images into the basic part and the detailed part, respectively, and then directly used the weighted average method to fuse the basic part, and used the deep learning framework to extract features for the detailed part, and finally reconstruct the fused image. In addition to extracting the feature information, Wen-Bo An et al. [15] constructed a supervised convolutional network to fully extract the complementary information of infrared and visible images, and the obtained fusion image better retained the details in the original image. In addition, end-to-end image fusion methods are also developing continuously. Ma et al. proposed the first image fusion method based on a generative adversarial network (GAN) [16], which transformed the fusion task into an adversarial learning process of infrared and visible image information retention, which opened up a new idea for the research of deep learning fusion methods. Zhang et al. [17] proposed a GAN image fusion algorithm based on the preservation of structural similarity. The experiments show that this method has improved various indicators compared with the previous methods. Algorithms based on deep learning provide a new direction for the development of image fusion. However, many training parameters and large amounts of data are two difficult problems for deep learning-based methods to solve.

To overcome the shortcomings of the above algorithms, a new adaptive robust algorithm that combines image decomposition and deep learning networks is designed in this paper. Different from the traditional two-scale decomposition algorithm, the proposed algorithm divides the image more carefully through three-scale decomposition, which lays a good foundation for subsequent fusion. Unlike sparse representation-based frameworks, the proposed algorithm does not require dictionary learning. Compared with deep learning-based models, the proposed algorithm only introduces a trained deep learning network for feature extraction, and therefore, it is not affected by the dataset size. At the same time, the algorithm can also give full play to the advantages of deep learning algorithms in extracting feature details. The main contributions of this paper are as follows:

(1) A three-scale decomposition method is proposed, through which the source images can be decomposed more finely, which lays a good foundation for subsequent fusion;

(2) The weighted least square (WLS) fusion model is improved, and the energy layer is fused by minimizing the WLS cost function. Through this method, the fusion image can fully extract the detailed information of the visible image without causing excess energy information in the infrared image;

(3) The fusion model of residual neural network (ResNet)-feature transfer is designed. By this method, the fusion detail layer can fully extract the contour structure information of the deep source image.

The remainder of this paper is organized as follows. Section 2 introduces the principles of rolling guided filtering and ResNet. Section 3 presents the algorithm model. Section 4 conducts the experiment and verifies the effectiveness of the proposed algorithm through qualitative and quantitative evaluation. The conclusions are in Section 5.

## 2. Theoretical Foundation

### 2.1. Rolling Guidance Filter (RGF)

RGF has scale-aware and edge-preserving properties. Therefore, it not only has a good ability to remove noise, but also can maintain the structure and edge characteristics of the source image. RGF consists of two main steps: small structure removal and edge restoration [18].

First a Gaussian filter is used to remove small structures, the image G filtered from the input image I can be expressed as:

(1) $$G=Gaussian\left(I,\sigma_{s}\right)$$

where $Gaussian\left(I,\sigma_{s}\right)$ represents the Gaussian filter and $\sigma_{s}$ represents the standard deviation as the scale parameter, through which the structural information, whose scale is smaller than the scale space, can be removed.

Guided filters [19] are then used for iterative edge recovery because it has better edge-preserving characteristics and higher computational efficiency than other filters. The second process is a step of iteratively updating the restored image $J^{t}$, and the initial image $J^{1}$ is a Gaussian smooth image G. The t-th iteration can be expressed as:

(2) $$J^{t+1}=GuidedFilter\left(J^{t},I,\sigma_{s},\sigma_{r}^{2}\right)$$

where $GuidedFilter\left(J^{t},I,\sigma_{s},\sigma_{r}^{2}\right)$ is the guided filter; $I,\sigma_{s}$ are the parameters in Equation (1); $J^{t}$ is the guided image; and $\sigma_{r}$ controls the distance weight. In this paper, we set $\sigma_{r}=0.05$. RGF is accomplished by combining Equations (1) and (2), which can be expressed as

(3) $$u=RGF\left(I,\sigma_{s},\sigma_{r},T\right)$$

where T is the number of iterations and u is the filter output.

### 2.2. Deep Residual Networks

With the development of deep learning, neural networks have been applied to various research fields. In general, the greater the number of network layers, the more information can be obtained. However, with the increase in the network layers, the problem of gradient descent will also occur, which will lead to the decline in the optimization effect. Without addressing this problem, He et al. [20] constructed a new neural network named ResNet in 2016, which improved the optimization ability and accuracy of the network by constructing multi-layer connections and residual representations. Subsequently, the ResNet network was widely used in the field of image processing and obtained great results on many occasions. Kang et al. [21] introduced a stagnation analysis method using a hierarchical ResNet, allowing the detection and recognition of four spatial steganography methods. Li et al. [22] designed an algorithm for image fusion using ResNet, which extracted deep features through the constructed network model, and finally achieved fusion. However, the currently used ResNet structure is still not deep enough, and the deep ResNet network is not widely used, especially in the field of infrared and visible image fusion.

**Figure 1:**
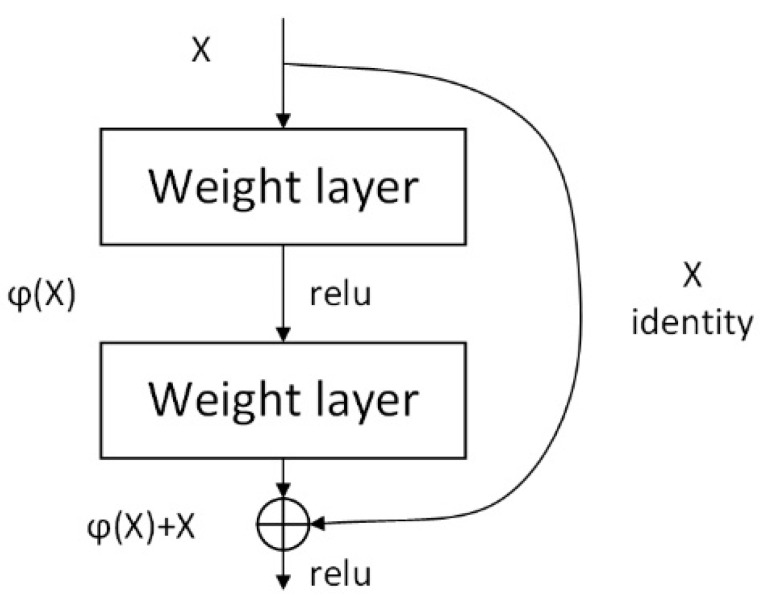
Residual Structure Module.

**Figure 2:**
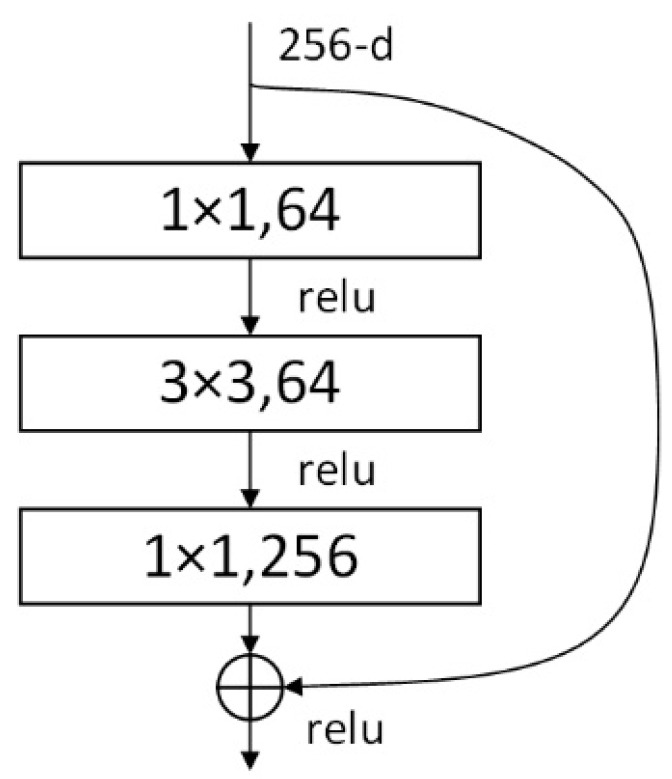
The ResNet bottleneck block structure.

The structure of ResNet is shown in Figure 1. X represents the input, φ(X) represents the identity mapping to the input X, and relu represents the activation correction of the network. φ(X)+X is the final output result. The ResNet residual structure used in this paper is shown in Figure 2. The main branch uses three convolutional layers, the first 1 × 1 convolutional layer is used to compress the channel dimension. The second is a 3 × 3 convolutional layer, and the third is a 1 × 1 convolutional layer to restore the channel dimension. Among them, the first two convolutional layers on the main branch have the same number of convolution kernels, and the third layer has four times as many.

The deep residual neural network is implemented through a shortcut connection, and the network is formed by an element-wise superposition. This structure not only does not add redundant variables and computation to the network, but also greatly improves the training effect and speed of the network. In addition, when the number of layers of the network structure increases, the degradation problem can also be well solved by such a structure. Therefore, ResNet152 is selected for feature extraction and fusion of infrared and visible images, and it is used to fuse the detail layer, which not only does not lose the structure details, but also can extract deeper information. It can preserve the structural features and details of infrared and visible images to the greatest extent. The trained ResNet152 deep feature mapping model is used for subsequent feature extraction processing, which effectively avoids the complex problem of network training and improves the efficiency of the algorithm.

## 3. Algorithmic Framework

**Figure 3:**
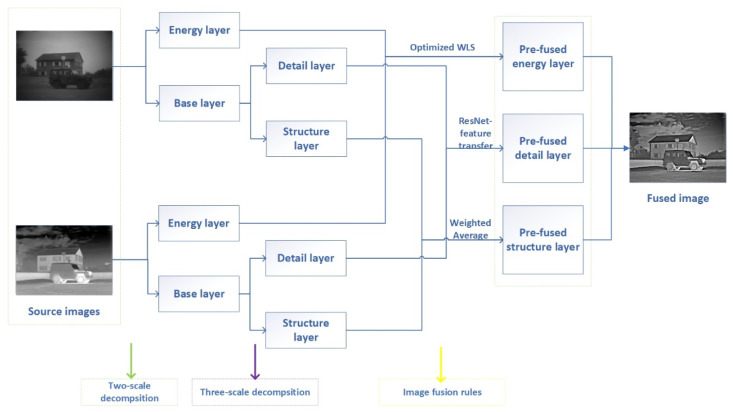
The scheme of image fusion algorithm.

A new image fusion model is constructed in this paper, as shown in Figure 3. Different from the traditional image fusion algorithm, first a three-scale decomposition scheme is adopted to decompose the image into three parts. Then, the characteristics of different components are analyzed, and different fusion rules for pre-fusion are designed. Finally, the final fused image is obtained by reconstructing the three pre-fused images. The specific implementation scheme is described in detail below.

### 3.1. Three-Scale Decomposition Scheme

To reduce the dependence on MST and improve the operation speed, an averaging filter is used to decompose the source image into a base layer that preserves the thermal-variant features of the target region and an energy layer that contains the gradient changes of texture details. Let $F_{a}$ denote an averaging filter of size 31 × 31, and $I_{ir}$ and $I_{vi}$ denote infrared and visible images, respectively. The base layers $B_{ir}$ and $B_{vi}$ can be summarized as:

(4) $$B_{ir}=I_{ir}*\;F_{a}$$

(5) $$B_{vi}=I_{vi}*\;F_{a}\;$$

where ∗ represents the convolution operator. Then, the energy layers $E_{ir}$ and $E_{vi}$ of the infrared image and the visible image can be expressed as:

(6) $$E_{ir}=I_{ir}-B_{ir}$$

(7) $$E_{vi}=I_{vi}-B_{vi}$$

After the base layer and the energy layer are obtained, since the amount of information in the base layer is still large, considering that the detail features and structural features can be fully extracted, the base layer is decomposed into the detail layer and the structure layer again by using RGF. The structural layers $S_{ir}$ and $S_{vi}$ can be expressed as:

(8) $$S_{ir}=RGF\;\left(B_{ir},\sigma_{s},\sigma_{r},T\right)$$

(9) $$S_{vi}=RGF\left(B_{vi},\sigma_{s},\sigma_{r},T\right)$$

where $\sigma_{s}=3$ and T=4. After obtaining the structure layer, the corresponding detail layer can be expressed as:

(10) $$D_{ir}=B_{ir}-S_{ir}$$

(11) $$D_{vi}=B_{vi}-S_{vi}$$

**Figure 4:**
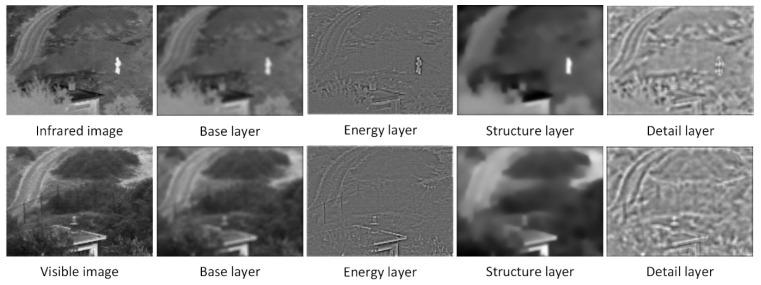
The results of three-scale image decomposition.

It can be seen from the results shown in Figure 4 that the constructed three-scale decomposition algorithm can effectively decompose the input image into the expected results. Among them, the energy layer contains most of the contour structure information, the structure layer contains brightness and contrast information, and the detail layer contains the remaining small amount of edge contour and detail information. It lays the foundation for the next design fusion strategy.

### 3.2. Fusion Scheme

According to the specific characteristics of different layers, the following different fusion schemes are designed.

#### 3.2.1. Energy Layer Fusion

Because the energy layer has more edge structure features and infrared energy features, the human visual system has a keen sense of energy. The traditional saliency detection algorithm can detect the information with prominent edge structure and obvious contrast difference, but it does not consider the infrared image and the visible image separately, and the detected information will be too rich in infrared information and insufficient extraction of visible light information. To overcome this defect, an optimized WLS energy layer fusion rule is proposed.

**Figure 5:**
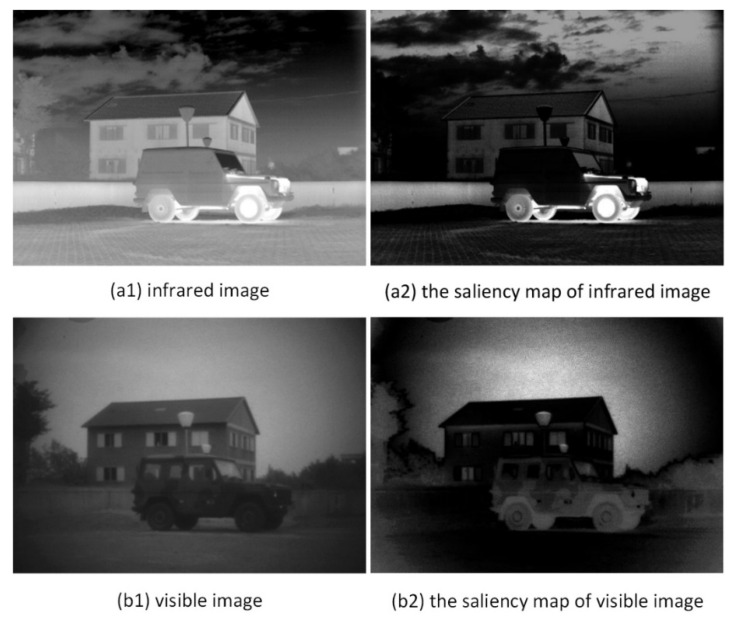
The saliency map of infrared and visible images.

First, saliency map and weight map need to be generated by a saliency detection method based on median filter and average filter. The saliency maps of infrared image $I_{ir}$ and visible image $I_{vi}$ are represented by $M_{1}$ and $M_{2}$, respectively, $F_{a}$ represents an average filter of size 31 × 31, and $F_{m}$ represents a median filter of size 3 × 3. Then, the saliency map can be expressed as Equations (13) and (14). Figure 5 shows a saliency map of a pair of infrared and visible images:

(12) $$M_{1}=\|I_{ir}*F_{a}-I_{ir}*F_{m}\|$$

(13) $$M_{2}=\|I_{vi}*F_{a}-I_{vi}*F_{m}\|$$

The edge structure information can be represented by the Euclidean distance of the difference between the average filtering and median filtering. In this process, without affecting the contour information, $F_{a}$ is used to weaken the sharp intensity change between adjacent pixels, and $F_{m}$ is used to achieve noise reduction. After the saliency map is obtained, appropriate weights should be assigned to the energy layers $E_{ir}$ and $E_{vi}$, respectively. $a_{1}$ and $a_{2}$ represent weights. In order to obtain more weights for places with rich detailed features, the weights are designed as follows:

(14) $$a_{1}=\frac{M_{1}}{M_{1}+M_{2}}$$

(15) $$a_{2}=\frac{M_{2}}{M_{1}+M_{2}}$$

where $a_{1},a_{2}\in \left[0,\;1\right]$. The initial fusion energy layer obtained by the saliency-based method is represented as $F_{E}^{*}$:

(16) $$F_{E}^{*}=a_{1}\times E_{ir}+a_{2}\times E_{vi}$$

However, $F_{E}^{*}$ obtained by this saliency detection method contains insufficient visible detail information and too much infrared energy information. To this end, inspired by the SWLS [9], the final energy layer fusion image $F_{E}$ is obtained by minimizing the following WLS cost function:

(17) $$\underset{\left(x,y\right)}{\sum }\left({\left(F_{E}\left(x,y\right)-F_{E}^{*}\left(x,y\right)\right)}^{2}+\frac{{\left(F_{E}\left(x,y\right)-E_{vi}\left(x,y\right)\right)}^{2}}{\sum_{\left(x,y\right)\in a_{\left(x,y\right)}}\left|E_{ir}\left(x,y\right)\right|+\beta }\right)$$

where (x,y) represents the location of the pixel. The role of ${\left(F_{E}\left(x,y\right)-F_{E}^{*}\left(x,y\right)\right)}^{2}$ is to make the final fusion energy layer $F_{E}$ structurally similar to the original fusion energy layer $F_{E}^{*}$. $\underset{\left(x,y\right)\in a_{\left(x,y\right)}}{\sum }\left|E_{ir}\left(x,y\right)\right|+\beta$ represents the coefficient of irrelevant infrared information, and the function is to reduce the redundant infrared energy information. β represents a minimal constant infinitely close to zero, which is set to $10^{-5}$ in this paper to prevent division by zero. $a_{\left(x,y\right)}$ is a convolutional window centered at position (x,y) to control the reduction in redundant information in infrared images of size 7 × 7. The function of ${\left(F_{E}\left(x,y\right)-E_{vi}\left(x,y\right)\right)}^{2}$ is to increase the important edge detail information of visible images. Finally, the fused energy layer $F_{E}$ is obtained by solving the above cost function. This process can effectively avoid information loss or information redundancy caused by the unified processing of infrared and visible images in traditional saliency detection algorithms.

#### 3.2.2. Detail Layer Fusion

**Figure 6:**
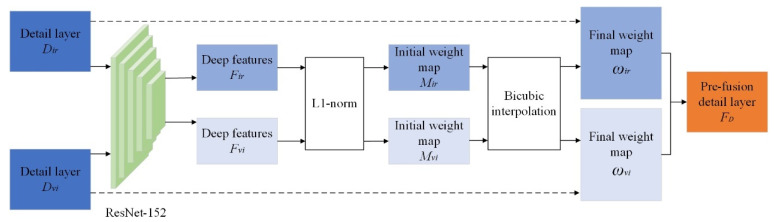
Detail layer fusion process.

Since the detail layer comes from the basic components of the source images, the detail contained in this layer is relatively weak, and it is difficult to fully extract its salient information by general image fusion methods. Therefore, the ResNet-feature transfer method is used to fuse the detail layers to obtain more detailed features. The specific fusion process is shown in Figure 6.

First, feature maps of image detail layers are extracted using ResNet152 [23]. Then, the weight map is obtained through the feature mapping operation in Equations (18) and (19). Finally, the detail layer fusion image $F_{D}$ is obtained by weight mapping and detail component reconstruction.

ResNet152 is a pre-trained network composed of 5 convolution blocks with a total of 152 weight layers. Therefore, the depth features $F_{ir}^{j,c}$ and $F_{vi}^{j,c}$ of the infrared and visible detail layer images output by the j-th (j∈{1,2,3,4,5}) convolutional block can be expressed as:

(18) $$F_{ir}^{j,c}=\varphi \left(D_{ir}\right)$$

(19) $$F_{vi}^{j,c}=\varphi \left(D_{vi}\right)$$

where c represents the number of channels in each deep feature layer. L1 regularization is performed on the depth features to obtain the initial weight map:

(20) $$M_{ir}^{\dot{j,*}}=\frac{\sum_{m=x-\theta }^{x+\theta }\sum_{n=y-\theta }^{y+\theta }\|F_{ir}^{j,c}{\left(m,n)\right\|}_{1}}{\theta \times \left(2\theta +1\right)}$$

(21) $$M_{vi}^{\dot{j,*}}=\frac{\sum_{m=x-\theta }^{x+\theta }\sum_{n=y-\theta }^{y+\theta }\|F_{vi}^{j,c}{\left(m,n)\right\|}_{1}}{\theta \times \left(2\theta +1\right)}$$

where θ=2 indicates that a matrix sparse operation with a stride of 5 × 5 is performed on the depth feature [22].

After obtaining two initial weight maps $M_{ir}^{\dot{j,*}}$ and $M_{vi}^{\dot{j,*}}$ through the two detail components $D_{ir}$ and $D_{vi}$, and bicubic interpolation is used to up-sample them. The initial weights are adjusted according to the size of source images. The weights of the final infrared and visible detail layer images are:

(22) $$\omega_{ir}^{j}=\frac{M_{ir}^{\dot{j,*}}\left(x,y\right)}{M_{ir}^{\dot{j,*}}\left(x,y\right)+M_{vi}^{\dot{j,*}}\left(x,y\right)}$$

(23) $$\omega_{vi}^{j}=\frac{M_{vi}^{\dot{j,*}}\left(x,y\right)}{M_{ir}^{\dot{j,*}}\left(x,y\right)+M_{vi}^{\dot{j,*}}\left(x,y\right)}$$

where $\omega_{ir}^{j}$ is the weight of the infrared detail layer image; $\omega_{vi}^{j}$ is the weight of the visible detail layer image; and (x,y) is the position of the pixel in the image.

The final fusion result of the detail layer is:

(24) $$F_{D}=\omega_{ir}^{j}\times D_{ir}\left(x,y\right)+\omega_{vi}^{j}\times D_{vi}\left(x,y\right)$$

#### 3.2.3. Structural Layer Fusion

The structural layer of the source image contains more overall structural information. Therefore, the weighted average strategy [24] is introduced to obtain the structure fusion image $F_{S}$:

(25) $$F_{S}=l_{1}S_{ir}\left(x,y\right)+l_{2}S_{vi}\left(x,y\right)$$

where $l_{1}$ and $l_{2}$ represent the weight values; and (x,y) are the pixel positions of the infrared structure layer image $S_{ir}$ and the visible structure layer image $S_{vi}$. In order to maintain the overall structure and light intensity information of the source images, and reduce useless information, the parameters are set as $l_{1}=l_{2}=0.5$.

The final fusion image F is:

(26) $$F=F_{E}+F_{D}+F_{S}$$

## 4. Experimental Results and Analysis

### 4.1. Experimental Setup

**Figure 7:**
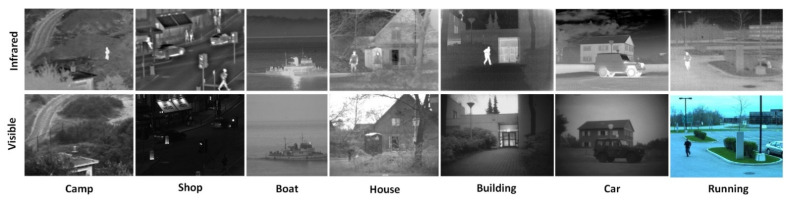
Seven pairs of images.

We used the infrared and visible image pairs in the public dataset to conduct experiments, and selected seven pairs of images for experimental display, as shown in Figure 7. Seven advanced algorithms including ResNet [22], CNN [25], GTF [26], IFEVIP [27], TIF [28], U2Fusion [29], and GANMcC [30] were selected to compare and verify them in the same experimental environment. All the experiments were accomplished using MATLAB R2018a 9.4.0 on a notebook PC with AMD Ryzen7 4800H with Radeon Graphics 2.90 GHz. In addition, six indicators were selected to quantitatively evaluate the fusion results, including entropy (EN) [31], edge information retention ($Q_{AB/F}$) [32], indicator proposed by Chen-Blum ($Q_{CB}$) [33], mutual information (MI) [34], structural similarity (SSIM) [35], and Visual Information Fidelity for Fusion (VIF). EN was used to measure the amount of information contained in the source image in the fusion image. $Q_{AB/F}$ utilizes local metrics to estimate how well salient information from source images is represented in fused images. $Q_{CB}$ is used as a human visual evaluation index to measure the quality of fused images. MI is used to measure the amount of information transferred from the source image into the fused image. SSIM is used to measure the structural similarity between the fused image and the source image. VIF can better reflect the degree to which the fusion result is consistent with the human visual perception. In summary, these metrics were chosen to evaluate the fused images obtained by the proposed algorithm from different perspectives.

### 4.2. Subjective Evaluation

**Figure 8:**
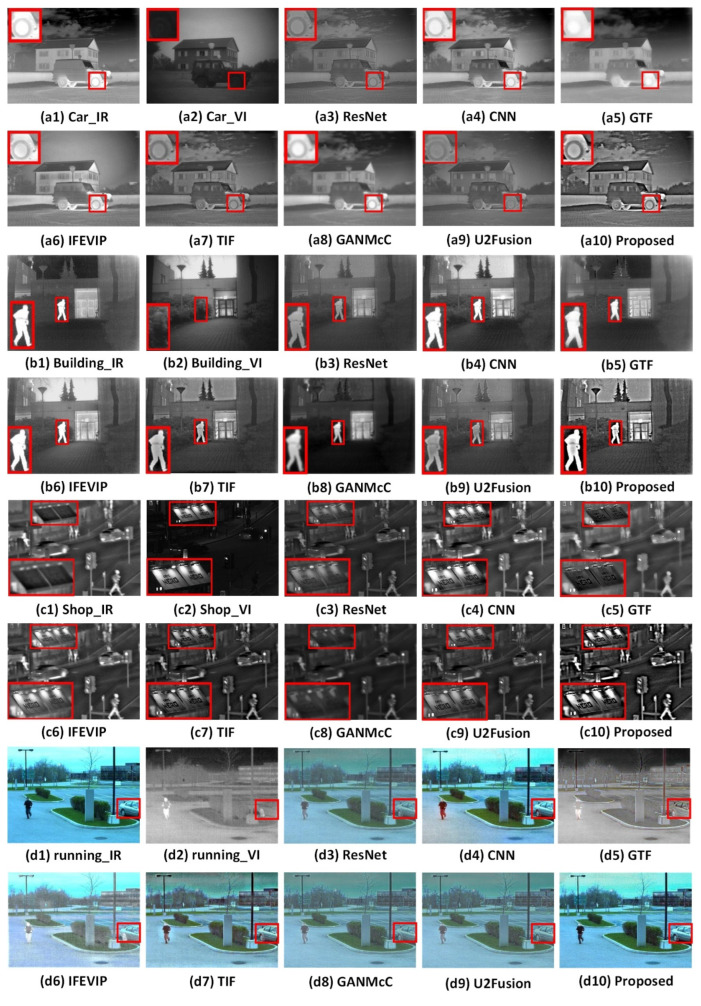
Fusion results of infrared and visible images.

**Figure 9:**
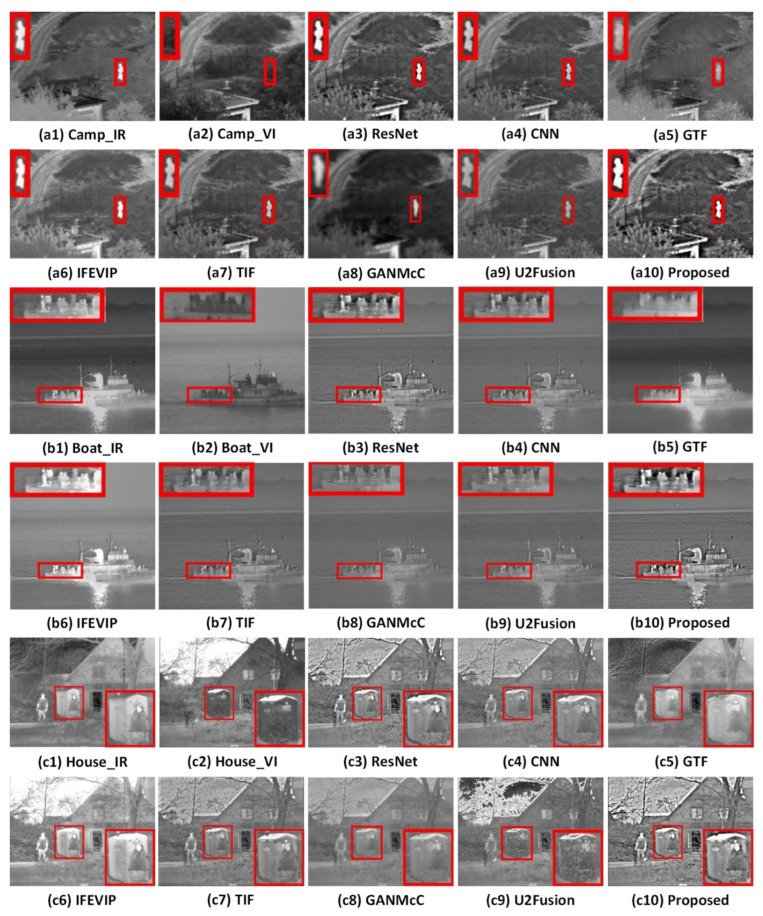
Fusion results of infrared and visible images.

The proposed algorithm was compared with seven state-of-the-art fusion algorithms, and the obtained results are shown in Figure 8 and Figure 9. Among them, the details we want to show in the picture are marked with red boxes and enlarged.

From Figure 8(a1–a10), it can be seen that our algorithm can effectively display the structural details’ information in the visible image and the energy and brightness information in the infrared image, especially for the ground and sky, and it is also more suitable for the brightness display of tires. However, the details shown by the GTF method are blurred. ResNet, CNN, and TIF methods can express most of the details in the source images, but there are still some places, such as window outlines, that are blurred. The IFEVIP method has a good demonstration of contrast expression, but the details of the sky in the picture are lost. The fusion results of the GANMcC method are blurry and less detailed information is displayed. Although the U2Fusion method can display a small amount of detailed information, it cannot clearly display the detailed information of the ground, sky, and other areas.

It can be seen from Figure 8(b1–b10) that the proposed algorithm has a good representation of the brightness of pedestrians, and has a good representation of the details of the ground, trees, and surrounding environment, and has a good outline representation. The ground details of the ResNet, GANMcC, and CNN methods are lost, and the tree details of the GTF method are lost. Although the IFEVIP and U2Fusion method expresses the detailed information well, its outline structure expression is not prominent.

As can be seen from Figure 8(c1–c10), the proposed algorithm not only maintains good details and contrast, but also can very clearly express the overall contours of vehicles, pedestrians, and roads. The ResNet and U2Fusion method shows poor brightness for billboards. The overall presentation of CNN, FTF, GANMcC, and IFEVIP is vague. Although the details of the TIF method are displayed, the contrast information is not well expressed.

In order to further verify the effectiveness of the proposed algorithm for the feature preservation of visible images, a pair of pictures taken during the day are shown in the Figure 8(d1–d10). In this case, the visible images have a better representation, while the infrared images have a poor description of the details. It can be seen from the figure that the proposed algorithm can better display the detailed information of the car, and can effectively extract the detailed information of the visible image and the contrast information in the infrared image, and the overall color is bright. However, the contrast methods are not good enough for the overall color representation of the image, and the fusion results of these methods have some artificial noise.

It can be seen from Figure 9(a1–a10) that the proposed algorithm can handle the structure outline and detail information of pedestrians, trees, and roads in the “Camp” scene well, and the contrast is high. The CNN and TIF methods do not adequately express the contrast of pedestrians. The ResNet and U2Fusion method outline structure is not clear. Fence details are not well expressed for GTF, GANMcC, and IFEVIP methods.

It can be seen from Figure 9(b1–b10) that the proposed algorithm expresses the details of the enlarged part very well, and the overall energy structure information is relatively complete. Although the ResNet, U2Fusion, and CNN methods express better details, the overall contrast is not high. The GTF, IFEVIP, GANMcC, and TIF algorithms are not good enough in the detail representation of the enlarged part.

From Figure 9(c1–c10), it can be seen that the proposed algorithm can display the detailed information of the phone booth, house, and trees well, and the house structure is prominent and the contrast is well expressed. The details of the ResNet method are well expressed, but its contour structure is not prominent. The CNN and TIF methods show a poor contrast between pedestrians and houses. The GTF, GANMcC, and IFEVIP methods are generally vague and have poor visual effects. Although the U2Fusion method can maintain the structural information of branches and houses, its overall feeling is discordant and the visual effect is poor.

To sum up, compared with the other five algorithms, the proposed algorithm can well express the energy information in infrared images and the details and contour structure information in visible images, and has good visual effects. In particular, the fusion results can show more detailed information than infrared images, such as houses, trees, etc., and can show more contrast information than visible images, such as clouds, ground textures, etc. This further demonstrates that the proposed fusion algorithm is effective. In addition, Table 1 shows the performance of each algorithm in five aspects: energy information, texture details, contour structure information, chromaticity information, and overall visual effect. It can be seen more intuitively that the proposed algorithm has better performance than other algorithms in all aspects. In Table 1, “+” represents better performance in this area, and “−“ represents poor performance. In addition, “+” and “−” do not explain the problem of the algorithm itself, but the relative advantages and disadvantages.

### 4.3. Objective Evaluation

**Figure 10:**
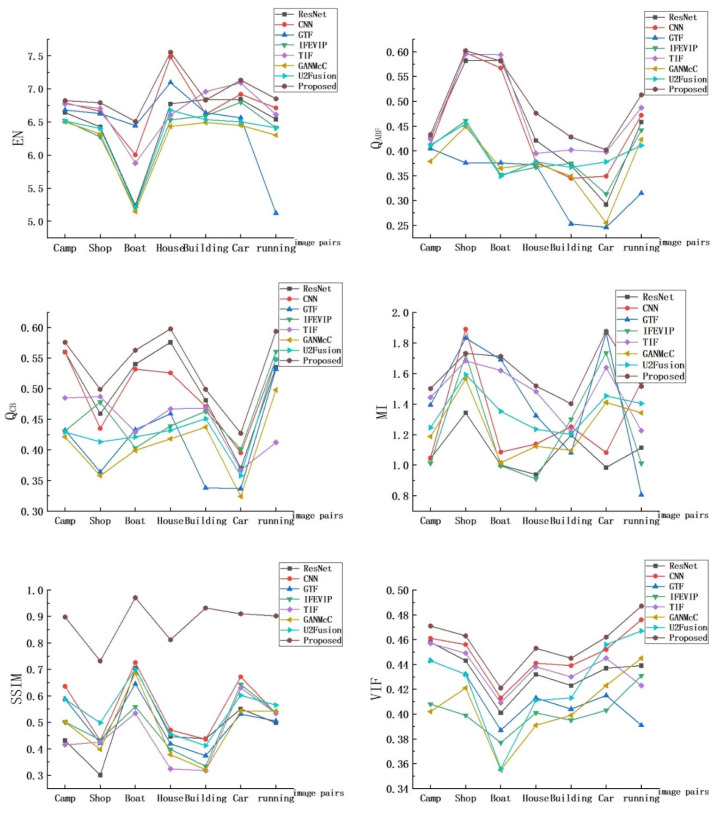
The objective evaluation results of the fusion results of the proposed algorithm and the other five algorithms.

The objective evaluation results of the fusion results of the proposed algorithm and the other five algorithms are shown in Figure 10. It can be seen that most of the indicators of the proposed algorithm are ranked in the front, which fully shows that the proposed algorithm has more outstanding performance in all aspects, and for $Q_{CB}$, SSIM, and VIF metrics, the proposed algorithm always performs optimally. Although TIF performs best in EN index in Building and $Q_{AB/F}$ in Boat, its overall performance is still worse than the algorithm proposed in this paper because its contour structure is not significant.

In addition, in order to enhance the reliability of the experimental results, we selected 21 pairs of image fusion results for quantitative experiments, and calculated the average value of each index of different algorithms. The results are shown in Table 2. The data in the table also show that the proposed algorithm has significantly higher objective evaluation index values than the other algorithms, which further proves the effectiveness of the proposed algorithm.

### 4.4. Computational Efficiency

The proposed algorithm and five contrasting algorithms are tested in the same experimental environment for the average time taken to fuse 21 pairs of images, and the results are shown in Table 3. Since the algorithm in this paper refers to the trained ResNet model, the algorithm runs much faster than the ResNet algorithm. In addition, since the proposed algorithm needs to perform three-scale decomposition and the fusion needs to be performed in steps, the speed of the proposed algorithm is slightly slower than the traditional algorithms GTF, IFEVIP, and TIF. However, it still has great advantages compared to the CNN, GANMcC, and U2Fusion algorithm. In future research, it is still an important research direction to continue to improve the performance of the algorithm to improve the computational efficiency.

## 5. Conclusions

In this paper, an infrared and visible image fusion algorithm based on three-scale decomposition and ResNet feature transfer is proposed. Different from other image decomposition methods, we propose a three-scale decomposition method, which decomposes the source image twice to obtain the energy layer, detail layer, and structure layer. Through this method, the source images can be decomposed more finely, which lays a good foundation for the subsequent fusion. In addition, the WLS fusion model is improved, and the energy layer is fused by minimizing the WLS cost function. Through this method, the fusion image can fully extract the detailed information of the visible image without causing excess energy information in the infrared image. Using the ResNet-feature transfer method to fuse the detail layers can fully extract the contour structure information of the deep source images. The structural layers are fused using a weighted average strategy. The experimental results show that the algorithm outperforms the other five comparison algorithms and has good visual effects.

## Figures and Tables

**Table 1:** Comparative results of all methods.

Feature	ResNet	CNN	GTF	IFEVIP	TIF	GANMcC	U2Fusion	Proposed
Energy	−	+	+	+	−	−	+	+
Texture detail	−	+	−	+	+	−	−	+
Contour Structure	−	−	−	+	+	−	+	+
Chromaticity	−	+	−	−	−	−	−	+
Visual effects	−	−	−	−	−	−	−	+

**Table 2:** Average quantitative evaluation results of fused images.

index	ResNet	CNN	GTF	IFEVIP	TIF	GANMcC	U2Fusion	Proposed
EN	6.46	6.746	6.675	6.319	6.668	6.426	6.451	6.94
QABF	0.446	0.441	0.338	0.38	0.468	0.417	0.423	0.487
QCB	0.498	0.487	0.394	0.436	0.451	0.413	0.422	0.527
MI	1.084	1.249	1.241	1.28	1.514	1.035	1.113	1.624
SSIM	0.358	0.656	0.513	0.508	0.493	0.341	0.398	0.901
VIF	0.432	0.443	0.416	0.397	0.438	0.401	0.419	0.452

**Table 3:** Compute efficiency of different methods.

Method	ResNet	CNN	GTF	IFEVIP	TIF	GANMcC	U2Fusion	Proposed
Time/s	20.73	23.16	2.91	1.34	1.03	**13.41**	**15.02**	3.16

## Data Availability

Not applicable.

## References

[1] Yang Z., Zeng S. (2022). TPFusion: Texture preserving fusion of infrared and visible images via dense networks. Entropy.

[2] Zhao Y., Cheng J., Zhou W., Zhang C., Pan X. Infrared pedestrian detection with converted temperature map. Proceedings of the 2019 Asia-Pacific Signal and Information Processing Association Annual Summit and Conference (APSIPA ASC).

[3] Arora V., Mulaveesala R., Rani A., Kumar S., Kher V., Mishra P., Kaur J., Dua G., Jha R.K. (2021). Infrared Image Correlation for Non-destructive Testing and Evaluation of Materials. J. Nondestruct. Eval..

[4] Burt P.J., Adelson E H., Fischler M.A., Firschein O. (1987). The Laplacian pyramid as a compact image code. Readings in Computer Vision.

[5] Toet A. (1989). Image fusion by a ratio of low-pass pyramid. Pattern Recognit. Lett..

[6] Toet A. (1989). A morphological pyramidal image decomposition. Pattern Recognit. Lett..

[7] Ren L., Pan Z., Cao J., Zhang H., Wang H. (2021). Infrared and visible image fusion based on edge-preserving guided filter and infrared feature decomposition. Signal Process..

[8] Kumar V., Agrawal P., Agrawal S. (2017). ALOS PALSAR and hyperion data fusion for land use land cover feature extraction. J. Indian Soc. Remote Sens..

[9] Ma J., Zhou Z., Wang B., Zong H. (2017). Infrared and visible image fusion based on visual saliency map and weighted least square optimization. Infrared Phys. Technol..

[10] Li H., Wu X.J., Kittler J. (2020). MDLatLRR: A novel decomposition method for infrared and visible image fusion. IEEE Trans. Image Process..

[11] Yang B., Li S. (2014). Visual attention guided image fusion with sparse representation. Opt.-Int. J. Light Electron Opt..

[12] Liu Y., Liu S., Wang Z. (2015). A general framework for image fusion based on multi-scale transform and sparse representation. Inf. Fusion.

[13] Chen G., Li L., Jin W., Zhu J., Shi F. (2019). Weighted sparse representation multi-scale transform fusion algorithm for high dynamic range imaging with a low-light dual-channel camera. Opt. Express.

[14] Li H., Wu X.J., Kittler J. Infrared and visible image fusion using a deep learning framework. Proceedings of the 24th International Conference on Pattern Recognition (ICPR).

[15] An W.B., Wang H.M. (2020). Infrared and visible image fusion with supervised convolutional neural network. Opt.-Int. J. Light Electron Opt..

[16] Ma J., Yu W., Liang P., Li C., Jiang J. (2019). FusionGAN: A generative adversarial network for infrared and visible image fusion. Inf. Fusion.

[17] Zhang D., Zhou Y., Zhao J., Zhou Z., Yao R. (2021). Structural similarity preserving GAN for infrared and visible image fusion. Int. J. Wavelets Multiresolut. Inf. Processing.

[18] Zhang Q., Shen X., Xu L., Jia J. Rolling guidance filter. Proceedings of the European Conference on Computer Vision.

[19] He K., Sun J., Tang X. (2012). Guided image filtering. IEEE Trans. Pattern Anal. Mach. Intell..

[20] Zhang H., Dana K. Multi-style generative network for real-time transfer. Proceedings of the European Conference on Computer Vision (ECCV) Workshops.

[21] Kang S., Park H., Park J.I. (2021). Identification of multiple image steganographic methods using hierarchical ResNets. IEICE Trans. Inf. Syst..

[22] Li H., Wu X., Durrani T.S. (2019). Infrared and visible image fusion with ResNet and zero-phase component analysis. Infrared Phys. Technol..

[23] Russakovsky O., Deng J., Su H., Krause J., Satheesh S., Ma S., Huang Z., Karpathy A., Khosla A., Bernstein M. (2015). Imagenet large scale visual recognition challenge. Int. J. Comput. Vis..

[24] Yang F., Li J., Xu S., Pan G. The research of a video segmentation algorithm based on image fusion in the wavelet domain. Proceedings of the 5th International Symposium on Advanced Optical Manufacturing and Testing Technologies: Smart Structures and Materials in Manufacturing and Testing.

[25] Liu Y., Chen X., Cheng J., Peng H., Wang Z. (2018). Infrared and visible image fusion with convolutional neural networks. Int. J. Wavelets Multiresolut. Inf. Process..

[26] Ma J., Chen C., Li C., Huang J. (2016). Infrared and visible image fusion via gradient transfer and total variation minimization. Inf. Fusion.

[27] Zhang Y., Zhang L., Bai X., Zhang L. (2017). Infrared and visual image fusion through infrared feature extraction and visual information preservation. Infrared Phys. Technol..

[28] Bavirisetti D.P., Dhuli R. (2016). Two-scale image fusion of visible and infrared images using saliency detection. Infrared Phys. Technol..

[29] Ma J., Zhang H., Shao Z., Liang P., Xu H. (2020). GANMcC: A generative adversarial network with multiclassification constraints for infrared and visible image fusion. IEEE Trans. Instrum. Meas..

[30] Xu H., Ma J., Jiang J., Guo X., Ling H. (2020). U2Fusion: A unified unsupervised image fusion network. IEEE Trans. Pattern Anal. Mach. Intell..

[31] Chibani Y. (2006). Additive integration of SAR features into multispectral SPOT images by means of the à trous wavelet decomposition. ISPRS J. Photogramm. Remote Sens..

[32] Xydeas C.S., Pv V. (2000). Objective image fusion performance measure. Mil. Tech. Cour..

[33] Yin C., Blum R.S. (2009). A new automated quality assessment algorithm for image fusion. Image Vis. Comput..

[34] Qu G., Zhang D., Yan P. (2002). Information measure for performance of image fusion. Electron. Lett..

[35] Zhou W., Bovik A.C., Sheikh H.R., Simoncelli E.P. (2004). Image quality assessment: From error visibility to structural similarity. IEEE Trans. Image Process..

